# Q&A: How do gene regulatory networks control environmental responses in plants?

**DOI:** 10.1186/s12915-018-0506-7

**Published:** 2018-04-11

**Authors:** Ying Sun, José R. Dinneny

**Affiliations:** 1 Department of Biology, Stanford University, 371 Serra Mall, Stanford, CA 94305 USA; 2 Department of Plant Biology, Carnegie Institution for Science, 260 Panama St, Stanford, CA 94305 USA

## Abstract

A gene regulatory network (GRN) describes the hierarchical relationship between transcription factors, associated proteins, and their target genes. Studying GRNs allows us to understand how a plant’s genotype and environment are integrated to regulate downstream physiological responses. Current efforts in plants have focused on defining the GRNs that regulate functions such as development and stress response and have been performed primarily in genetically tractable model plant species such as *Arabidopsis thaliana*. Future studies will likely focus on how GRNs function in non-model plants and change over evolutionary time to allow for adaptation to extreme environments. This broader understanding will inform efforts to engineer GRNs to create tailored crop traits.

## Question 1: What is a gene regulatory network?

**Fig. 1.**
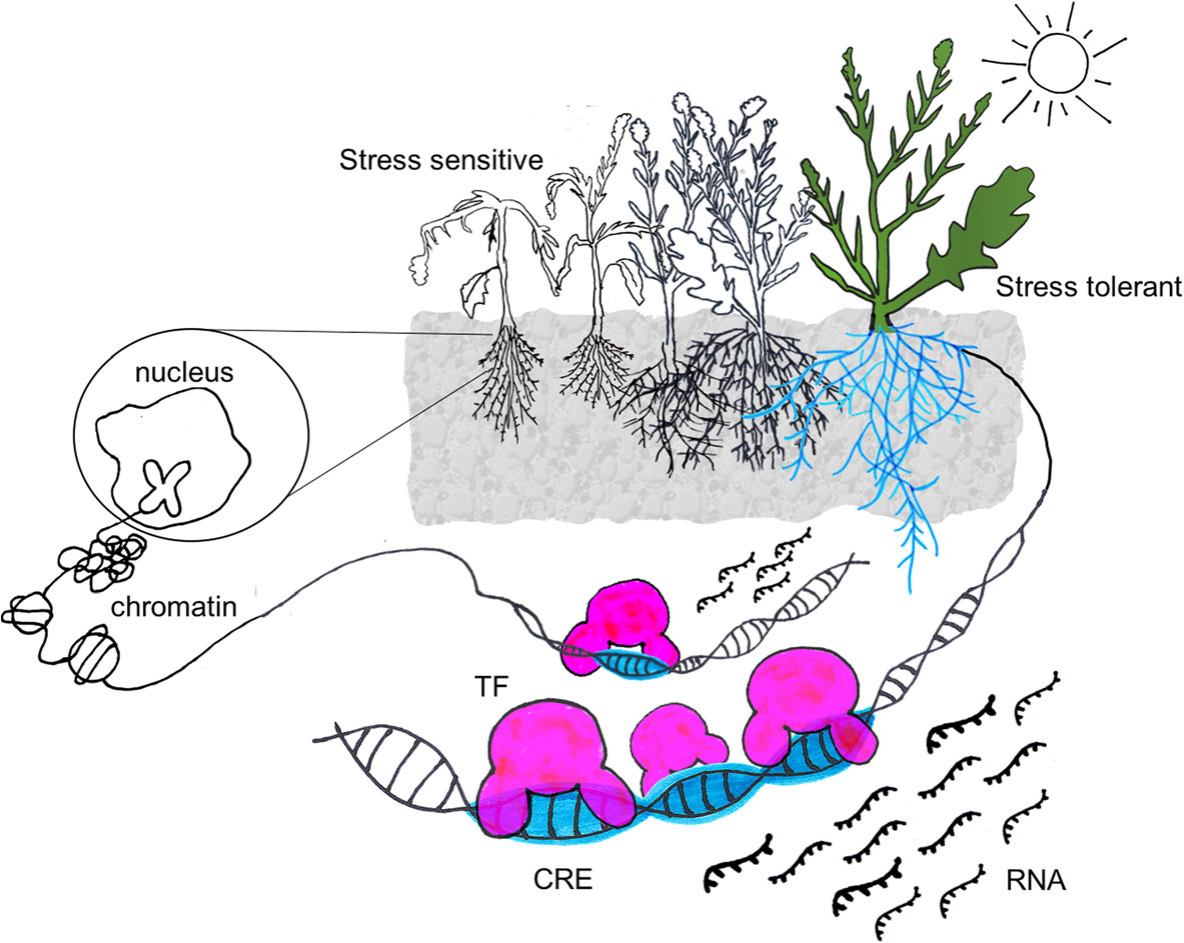
Plants exposed to stress in the environment elicit changes in the expression of genes mediated by transcription factors (*TF*). Interactions between TF and their associated *cis*-regulatory element (*CRE*) regulate the abundance of RNA expressed from different genes. Combinations of TF–CRE interaction lead to the establishment of gene regulatory networks (GRNs). Variation in the GRN may lead to different responses of the plants to the environmental stress

A gene regulatory network (GRN) is composed of molecular regulators such as transcription factors (TFs) that bind to short, non-coding DNA sequences called *cis*-regulatory elements (CREs), which are typically located in the promoter region of a gene [1, 2]. Transcriptional regulators and their target genes form an interconnected regulatory network that integrates endogenous and environmental cues into changes in gene expression (Fig. 1) [3–5].

## Question 2: How will studying GRNs improve our understanding of plant biology?

GRNs are often composed of thousands of connections between TFs and target genes that together, regulate many cellular functions. GRNs are complex and can be differentially regulated across tissue types and organs during plant develop or environmental acclimation [6–8]. Such complexity can be difficult to tackle experimentally because each part of the GRN requires many experiments to characterize each part of the GRN. To understand the function of this network in the regulation of a process of interest, it can be useful to identify points within the network that are most critical. These points in the network are frequently interaction hubs that target, or are targets of, many other genes and proteins in the network. Functionally characterizing these points within GRNs will likely improve our understanding of the biology of the plant [9].

## Question 3: Why have most GRN studies in plants utilized *Arabidopsis* as a model?

Many studies to understand the function of GRNs have used the model plant *Arabidopsis thaliana* because of the substantial genetic resources generated by the research community [10]. *Arabidopsis* is distributed across a wide range of habitats around the globe. Its genetic diversity has contributed to its ability to adapt to local environments. The genetic diversity within *Arabidopsis* provides an opportunity for understanding how the evolution of GRNs could contribute to environmental adaptations [11, 12]. To date, the genomes of over 1000 natural accessions of *Arabidopsis* from around the world have been sequenced and can be used to profile the functional effects of sequence variation on plant physiology [13]. Furthermore, relatives of *Arabidopsis* have been used to understand how environmental response traits may have evolved [14]. Currently, more than 285 plant genomes have been sequenced and span more than nine families of vascular plants, including 14 in the Brassicaceae family to which *Arabidopsis* belongs [15, 16]. Characterizing gene content within plant genomes has revealed that plants have a large number of TF families, suggesting that they have extensive GRNs like other complex eukaryotes [17].

## Question 4: How do we currently study GRNs?

Recent studies of GRNs have focused on defining the genes and proteins that make up the network and the molecular interactions that regulate those genes and proteins. This has been facilitated by the establishment of genome-wide datasets including whole genome sequences and transcriptomic profiling in different tissues and conditions. More recently, high-throughput assays to profile TF binding site preference and chromatin structure has established how TF-DNA interaction influences the expression of genes within a GRN [18]. Construction of a GRN can also focus on TFs and target genes that likely function together in a specific biological pathway. For example, a GRN associated with secondary cell wall biosynthesis was constructed using yeast-1-hybrid assays and led to the discovery of stress-responsive changes in wall composition [19]. Additionally, the global-scale analysis of TF-target interactions using ChIP-Seq established an extensive GRN acting downstream of 21 TFs controlling response to the hormone abscisic acid (ABA) [20]. In vitro biochemical assays such as DNA affinity purification and sequencing (DAP-Seq) have also been used to broadly survey the direct genomic targets of several hundred TFs [21].

Additional computational tools are being developed so GRNs can be utilized to understand dynamic regulatory processes in plants. For example, the Environmental Gene Regulatory Influence Network (EGRIN) uses an algorithm to incorporate genome-scale transcriptome data from controlled and agricultural field experiments, and chromatin accessibility measurements into a model that predicts TF activity in response to changing environmental conditions [22]. Integrating multiple layers of regulation improves the predictive power of GRNs and can identify potential mechanisms for crosstalk between pathways [23]. Incorporating tissue and developmental stage-specific transcriptome data identified TF nodes that function in both stress and developmental signaling pathways [24]. These tools have been powerful in determining how groups of genes within GRNs are being regulated together and improves our knowledge of how genotype determines phenotype.

## Question 5: How does genetic variation affect the architecture of GRNs?

Genetic variation within a species can have important effects on a GRN; changes in the coding sequence of a TF can change binding site preference, and sequence variation in promoters can result in the gain or loss of CREs [25]. To understand how sequence variation ultimately leads to differences in downstream physiology, GRNs can be constructed to include sequence differences that exist within a species or across species [26]. Analysis of genetic variation in *Arabidopsis* has revealed a greater number of polymorphisms in the promoter regions of drought and cold responsive genes than genes with other functions, suggesting that differences in CRE composition may be involved in local adaptation to environmental stress [27]. It is likely that comparing GRNs between species will help identify points in the network where genetic variation contributes to functional differences in gene regulatory mechanisms [26].

## Question 6: How can GRNs be experimentally manipulated?

GRNs can be experimentally manipulated using gene knockouts, gene silencing, and editing approaches, such as viral-induced gene silencing (VIGS) and clustered regularly interspaced short palindromic repeats (CRISPR)/CAS9 system, respectively. Functional characterization of genes in GRNs through mutational analysis can help to validate the relationships between TFs, their target genes, and the phenotypes GRNs govern. The development of the (CRISPR)/CAS9 system has also greatly improved the specificity, efficiency, and throughput of genome editing [28]. Recently, the CAS9 system was used to create different promoter isoforms and this led to novel inflorescence architectures that affected tomato yield [29]. Parts of GRNs can also be reconstituted in heterologous systems to identify the necessary components needed to compose a GRN [30]. This has been effectively demonstrated for the auxin signaling pathway, where engineered yeast are able to induce target genes in response to exogenously supplied hormone [31, 32].

## Question 7: What are the future opportunities for understanding GRNs?

Our ability to predict the function and dynamical states of GRNs will be enhanced by improvements in computational modeling. Using Bayesian networks, GRNs can be inferred with small false positive rates. Markov models allow stochastic GRN dynamics to be studied. Additionally, neural models with higher learning rate and better predictive power are being used to study all possible gene-to-gene regulatory interactions. The Extreme learning machine is able to reconstruct predictive GRNs from only transcriptomic datasets [33]. Future questions the field may address will include: What datasets are needed to build predictive GRNs? Based solely on the genome of a plant, can we predict the adaptive traits the plant has? How do GRNs change over evolutionary time and during domestication, and can we domesticate plants more efficiently through an understanding of the GRN?

## Question 9: Where can I find more information?

- Gene regulatory networks [1–3, 26, 34, 35]
- The diversity of TF families in plants [17]
- Current updates on synthetic biology [30, 36, 37]
- Abiotic stress and NaCl stress in plants [38–43]
- Effects of the environment on root systems [44, 45]
- ABA and signals involved in plant stress response [46]
- Plant plasticity and evolution of tolerance traits [47, 48]
- Impact of CRE on stress response [49]
- Spatio-temporal GRNs [6, 22, 50–54]
- Halophytes and stress tolerant plants [55–60]
